# Conjugation effect of amine molecules in non-aqueous Mg redox flow batteries

**DOI:** 10.1039/d5sc04532k

**Published:** 2025-07-31

**Authors:** Yunan Qin, Vaidyanathan Sethuraman, Seong-Gyu Choi, Richard Gonzalez, Chengxiang Chen, Lei Cheng, Chao Luo, Tao Gao

**Affiliations:** a Department of Chemical Engineering, University of Utah Salt Lake City UT USA taogao@chemeng.utah.edu; b Chemical Sciences Division, Oak Ridge National Laboratory Oak Ridge TN USA; c Department of Chemical, Environmental and Materials Engineering, University of Miami Coral Gables FL USA cxl1763@miami.edu

## Abstract

Nonaqueous magnesium redox flow batteries (Mg RFBs) are attractive for low-cost, high-energy-density and long-cycle-life stationary energy storage applications. However, state-of-the-art cathode redox-active molecules suffer from low solubility and low redox potential. Herein, we screened a range of cathode redox-active molecules and identified amine molecules as optimal to couple with the Mg anode. The properties of amine derivatives and their performances were collected to establish the correlation between molecular structures and electrochemical performances. The redox potential and solubility of these amine molecules were influenced by the π-conjugated and non-conjugated structures of amine derivatives. Density functional theory (DFT) simulations and the inverse aromatic fluctuation index (FLU^−1^) verified that conjugation had an important role in stabilizing the molecule and increasing its redox potential. Notably, tris[4-(diethylamino)phenyl]amine (TDPA) achieved the highest theoretical energy density (∼120 Wh L^−1^) due to its high solubility (∼0.9 M) and voltage (∼2.5 V *vs.* Mg/Mg^2+^). We also demonstrated that ether solvents were crucial for stable, high-solubility catholytes, while bulk anions did not affect the redox potential of these p-type molecules. In a Mg-amine RFB configuration, the battery delivered 2.50 V, a specific discharge capacity of 106.5 mAh g^−1^, an initial coulombic efficiency of 90.74%, and a capacity retention of 93.88% after 150 cycles.

## Abstract 2

Nonaqueous magnesium redox flow batteries (Mg RFBs) with amine cathodes provide low-cost, high-energy-density, long-cycle-life energy storage. The redox potential and solubility of these amines are strongly shaped by their degree of conjugation.

## Graphical abstract

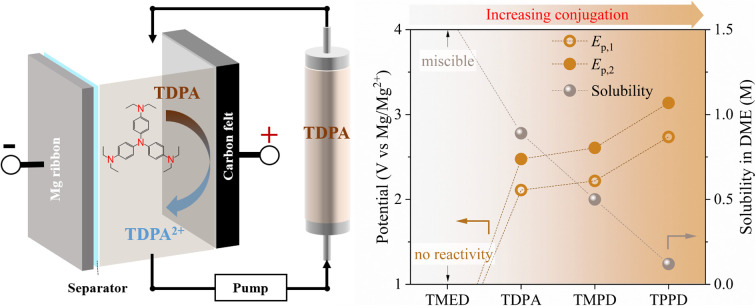

## Introduction

Owing to the limited resources and negative environmental impacts (CO_2_ emissions) of fossil fuels, it is necessary to explore renewable energy sources for electricity production.^1,2^ Such a transition requires efficient energy storage solutions to address the intermittency of renewable energy. Among different storage technologies, electrochemical energy storage (or batteries) has gained broad attention.^3,4^ According to the International Energy Agency (IEA), energy storage must grow sixfold by 2030 to support a threefold increase in global renewable energy capacity, with batteries expected to account for 90% of this expansion.^5^ This increase reflects the importance of electrochemical energy storage. Li-ion batteries (LIBs) are the most studied and deployed energy storage technology, but they suffer from supply chain challenges and high cost.^6,7^ Therefore, there is an urgent need for alternative energy storage technologies given the huge demands for affordable energy storage for grid resilience.

Redox flow batteries (RFBs) have the potential to meet these requirements. Redox-active molecules are dissolved in liquid electrolytes and pumped through external tanks to electrodes for electrochemical reactions.^8^ This unique cell structure benefits readily and independently scalable energy and power, as well as other advantages, such as fast solution reaction kinetics, minimal phase transformation, long cycle life and safety.^9^ Based on the solvent used in the electrolyte, RFBs can be divided into aqueous and nonaqueous types. To date, commercial flow batteries are aqueous, such as all-vanadium batteries and hybrid zinc batteries.^10,11^ However, the operation voltage of these aqueous RFBs is usually <1.5 V due to electrochemical water-splitting.^12^ The low voltage limits the power density and energy density of aqueous RFBs. Moreover, the use of high-cost and toxic materials such as vanadium in these aqueous RFBs poses challenges to the environment and energy sustainability. To address these challenges and achieve high-power-density and high-energy-density energy storage systems, the development of high-voltage nonaqueous RFBs offers promising opportunities.^13^

Among various nonaqueous RFBs, Mg RFBs stand out.^14–16^ The motivation in novel Mg RFBs is rooted in two unique advantages of Mg electrochemistry. The first advantage is high voltage and energy density. Mg anodes have low redox potential (−2.37 V *vs.* standard hydrogen electrode) and a high volumetric capacity (3833 mAh cm^−3^). In addition, Mg metal is denser than Li, offering twice the capacity at the same volume. The second advantage is a highly reversible and stable anode. The side reaction between the Mg metal anode and the electrolyte is minimal in ethereal organic electrolytes, leading to reversible Mg deposition/stripping for up to 2500 cycles.^17^ Such an advantage promises a stable anode/electrolyte interface and ensures a long cycle life.

Organic redox-active molecules are of particular interest as charge carriers in RFBs, thanks to their natural abundance, molecular diversity, and structural tunability.^18,19^ Their functional groups can be readily modified to adjust physical and electrochemical properties, such as solubility, chemical stability, redox potential, and kinetics.^20^ So far, most organic cathode molecules have been explored in acetonitrile (AN)-based electrolytes because they can provide high conductivity and wide potential windows.^21^ However, AN is not compatible with an Mg metal anode due to its high reactivity. Another design challenge for organic redox-active molecules in Mg RFBs is rooted in the instability of the soluble cathode and/or charged cathode with the Mg anode. Therefore, it is important to find suitable organic material that is compatible with an Mg anode and ethereal electrolytes. Considering that energy density is equal to *n* × *C* × *F* × *V* (where *n* is the number of transferred electrons, *C* is the concentration of redox-active molecule, *F* is Faraday's constant, and *V* is the cell voltage), requirements for high-performance organic cathode molecules also include: (1) multi-electron redox reactions; (2) high solubility; (3) high voltage. In consequence, it is crucial to develop a high-voltage and high-capacity catholyte that is compatible with an Mg metal anode to advance nonaqueous Mg RFBs.

**Fig. 1:**
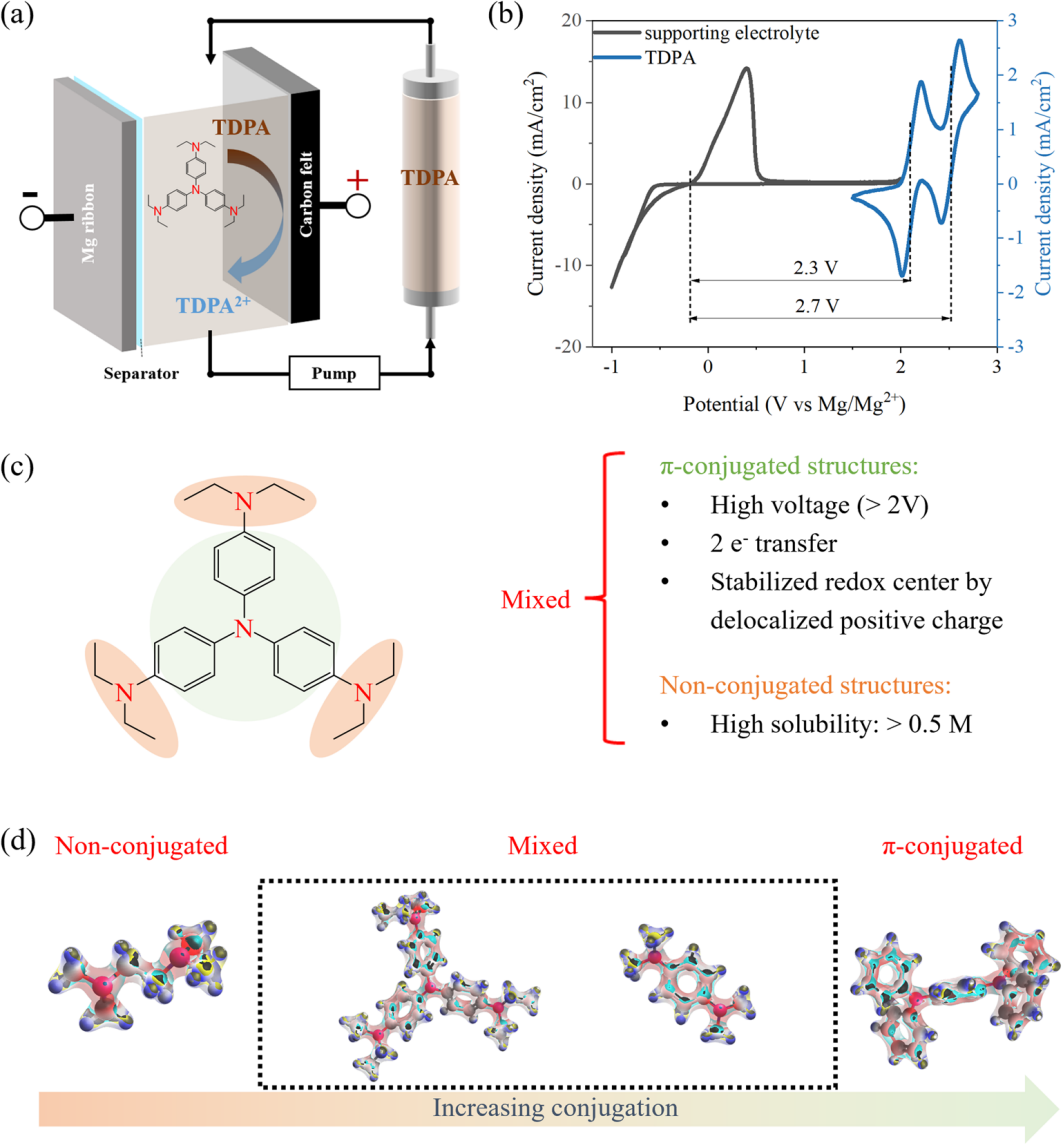
Scope. (a) Mg RFBs (schematic). (b) CV of the Mg anode reaction and amine-based catholyte reaction. (c) Design principle of amine molecules as the catholyte for Mg RFBs. (d) Electrostatic potential based on the electron density from natural bond orbital analysis^22^ in the Gaussian 16 (ref. 23) package. Figures were rendered using Avogadro software.^24^ Red indicates higher charge density, and blue indicates lower charge density.

In this work, we first comprehensively examined a wide range of organic molecules with different redox-active moieties in an Mg battery electrolyte (Table S1), identified the best compatible redox-active molecule in nonaqueous Mg RFBs (Fig. 1a and b), and obtained the key parameters to design a high-performance catholyte (Fig. 1c and d). After preliminary screening, an amine-based molecule stood out due to its high voltage and high compatibility with the Mg electrolyte. As such, to identify the most suitable amine molecules for Mg RFBs, we examined the multiple design freedoms of amine-based catholytes in RFBs, including the influence of redox-active moieties, non-conjugated and π-conjugated structures, and supporting electrolytes. By combining electrochemical tests with spectroscopic characterizations, we evaluated the performance of selective amine-based catholytes in a three-electrode cell and lab-made flow cell and correlated their performance with structures and properties (solubilities, transports, kinetics and stabilities). Density functional theory (DFT) simulations and the average inverse aromatic fluctuation index (FLU^−1^) were conducted to understand the impact of conjugation degree on the oxidation potential. Hansen solubility parameters (HSPs) were employed to analyze how molecular conjugation degree and solvent polarities influence the solubility behavior of organic molecules. Fundamental knowledge of the chemistry–property–performance correlation of this family of catholytes in various supporting electrolytes and metal anodes was obtained to set the foundation for further catholyte engineering.

## Results

### Screening of organic redox moieties for Mg RFBs

**Fig. 2:**
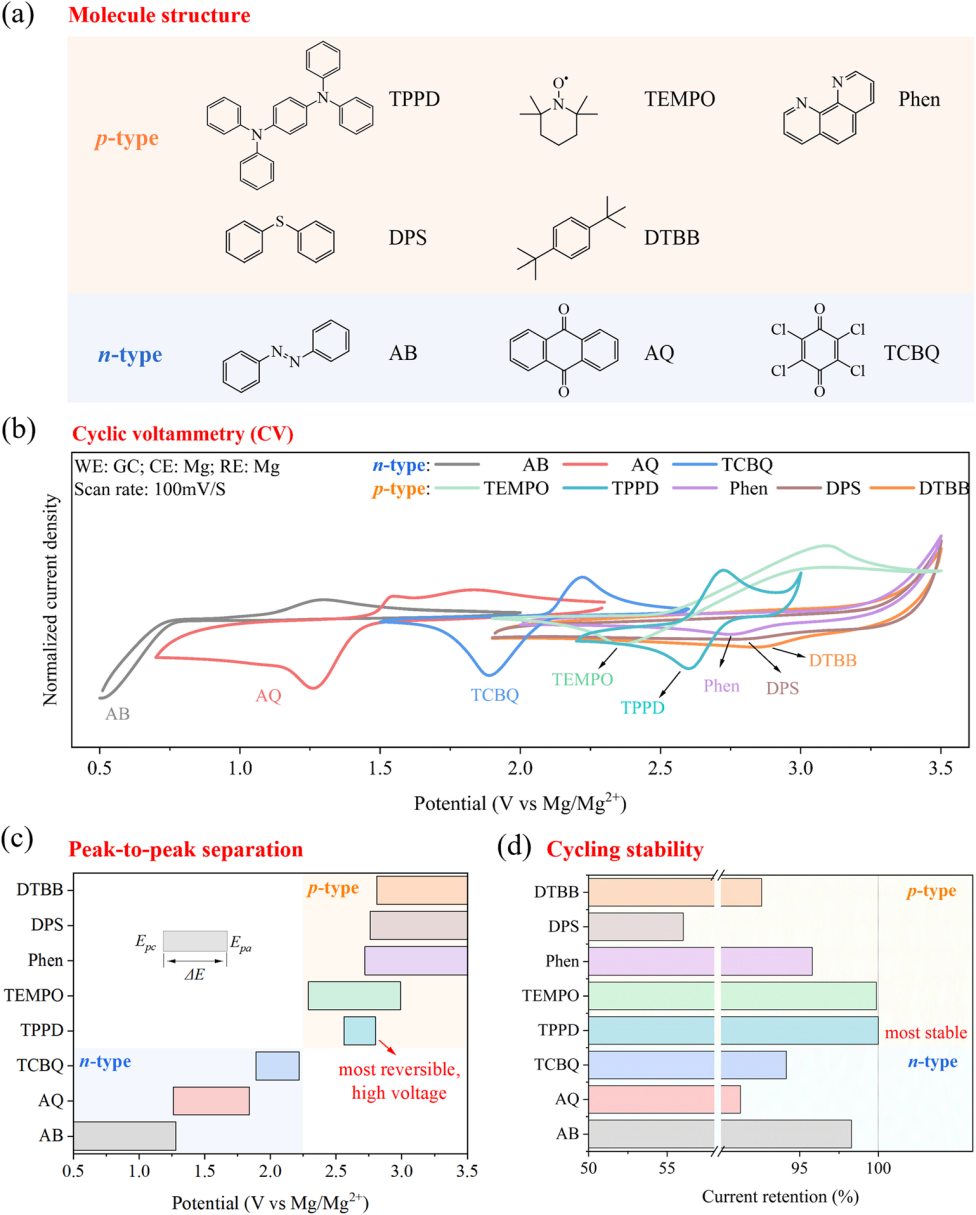
Screening of organic redox moieties for Mg RFBs. (a) Molecular structure of organic molecules with different redox-active moieties. (b) CV profiles, (c) peak-to-peak separation (Δ*E*) from CV results, and (d) cycling stabilities of these molecules in the Mg electrolyte. The concentration of active materials was set at 1 mmol/l (1 mM). Due to variations in solubility among these molecules, this concentration was chosen to ensure a consistent comparison under identical conditions. The Mg electrolyte was prepared by dissolving LiTFSI (0.5 M), Mg powder (0.25 M), and MgCl_2_ (0.5 M) in dimethoxyethane (DME).

To screen the best compatible redox moieties in a Mg electrolyte system, we examined the common organic molecules with different redox centers. These molecules included azobenzene (AB, azo), anthraquinone (AQ, quinones), tetrachloro-1,4-benzoquinone (TCBQ, quinones), *N*,*N*,*N*′,*N*′-tetraphenyl-1,4-phenylenediamine (TPPD, amine), (2,2,6,6-tetramethylpiperidin-1-yl)oxyl (TEMPO, nitroxide radical), 1,10-phenanthroline (Phen, pyridine), diphenyl sulfide (DPS, sulfide) and 1,4-di-*tert*-butylbenzene (DTBB, dialkoxybenzene) (Fig. 2a and Table S1). Most of these molecules have been demonstrated to exhibit favorable redox activity in other types of flow batteries, while their electrochemical behavior in the context of Mg-based electrolytes deserves investigation.

Azobenzene (AB), composed of two phenyl rings connected by an azo functional group, has demonstrated promising potential for non-aqueous RFBs owing to its high solubility and excellent electrochemical stability.^25^ Quinones and their derivatives, which contain two or more carbonyl groups connected by conjugation structures, have been widely studied owing to their highly reversible electrochemical redox reactions and fast kinetics.^26,27^ Herein, two types of quinone were selected: one was AQ and the other was TCBQ. AQ consists of an anthracene structure with two carbonyl groups (C=O) at *para* positions. This creates an extended aromatic and conjugated system, enhancing its stability and electrochemical reversibility. TCBQ has a single benzene ring substituted by two carbonyl groups at *para* positions and four chlorine atoms, which significantly influence its electronic properties by introducing strong electron-withdrawing effects.^28,29^ Amines are a class of molecules characterized by C–N bonds. Herein, we focused specifically on aromatic amines, composed of a phenyl group bonded directly to amine groups. Aromatic amines exhibit several desirable properties for redox flow applications, including ease of functional-group modification (enabling “tailored” adjustment of redox potential and solubility) and low reorganization energy when converting between neutral and radical forms.^30^ In this section, TPPD was selected as a representative aromatic amine molecule. TEMPO is one of the most widely used nitroxide radicals due to its exceptional stability, which arises from electron delocalization between nitrogen and oxygen atoms, as well as the steric protection provided by the four surrounding methyl groups.^31,32^ TEMPO was selected to assess its compatibility with Mg-based electrochemistry. 1,10-phenanthroline (Phen) is commonly used in iron and cobalt redox flow batteries because it can form complexes with metals, thus creating new redox couples.^33,34^ Herein, we test whether Phen molecules could similarly form complexes with Mg, potentially exhibiting redox activity. In addition, due to the structural similarity between Phen and amine molecules, which both contain nitrogen atoms bonded to aromatic rings, Phen can serve as a reference compound for comparison with amines. DPS (sulfide compound) and DTBB (dialkylbenzene derivative) are aromatic compounds containing benzene rings. In DPS, two phenyl groups are bridged by a sulfur atom, whereas DTBB features *tert*-butyl substituents on the benzene ring. These molecules were selected to investigate whether sulfur atoms or alkyl chains attached to aromatic systems could exhibit reversible redox activity in Mg-based electrolytes.

According to the different electric charges carried by the redox centers during electrochemical reactions, these organic molecules can be divided into p-type and n-type active materials.^35^ AB, AQ, and TCBQ belong to n-type active materials, carrying negative charges during charge/discharge, while the other molecules belong to p-type active materials, carrying positive charges during charge/discharge. Cyclic voltammetry (CV) was conducted to test the redox activities and potentials of these molecules in the Mg electrolyte (Fig. 2b). As summarized in Fig. 2c and Table S2, p-type molecules showed a higher redox potential (>2.6 V *vs.* Mg/Mg^2+^) than n-type molecules, which is consistent with the literature.^36^ Among these molecules, only AQ, TCBQ, TPPD and TEMPO showed clear pairs of redox peaks, indicating that quinones, amines and nitroxide radical were compatible in Mg chemistry. Their reaction mechanisms are given in Fig. S1. Notably, although TEMPO displayed reversible redox peaks, it showed a large peak-to-peak separation. As previously reported, Li^+^ exhibits a strong solvation interaction with the N–O˙ site of TEMPO, leading to the formation of a counterion–organic solute pair.^37^ Additionally, a peak-to-peak separation of approximately 0.2–0.6 V for TEMPO in a NaClO_4_–PC electrolyte has been reported.^38^ Therefore, the large peak-to-peak separation observed for TEMPO could be attributed to the presence of cations (Mg^2+^ and Li^+^) in the electrolyte, which deserves investigation in future work. Reversible redox peaks were not observed for the azo, sulfide, or dialkylbenzene compounds in the Mg electrolyte.

Among these high-potential molecules, TPPD showed the smallest peak-to-peak separation (Δ*E*), indicating that its redox reaction was most reversible. All the organic molecules, upon oxidation, formed radicals or charged species that were stabilized by their conjugated structures (phenyl or N–O bond), resulting in the enhanced stability of the charged molecules. The electrochemical stabilities of these organic molecules (Fig. 2d) were evaluated by examining the peak current decay during a 300-cycle CV scan and quantified by the peak current ratio of the 300th cycle to the first cycle (*I*_p,300_/*I*_p,1_) (Fig. S2). TPPD showed the highest stability (100%) compared with the other molecules, demonstrating the stable electron–transfer reaction of the amine group.

In general, the amine group was the most promising redox-active molecule for Mg RFBs owing to its high redox potential, high reversibility and high stability. In the subsequent study, the influence of its molecular structure on its properties (thermodynamics, solubilities, kinetics, transports and stabilities) was investigated to determine the best structure for amine catholyte design in Mg RFBs.

### Establishment of chemistry–properties–performance relationship

**Fig. 3:**
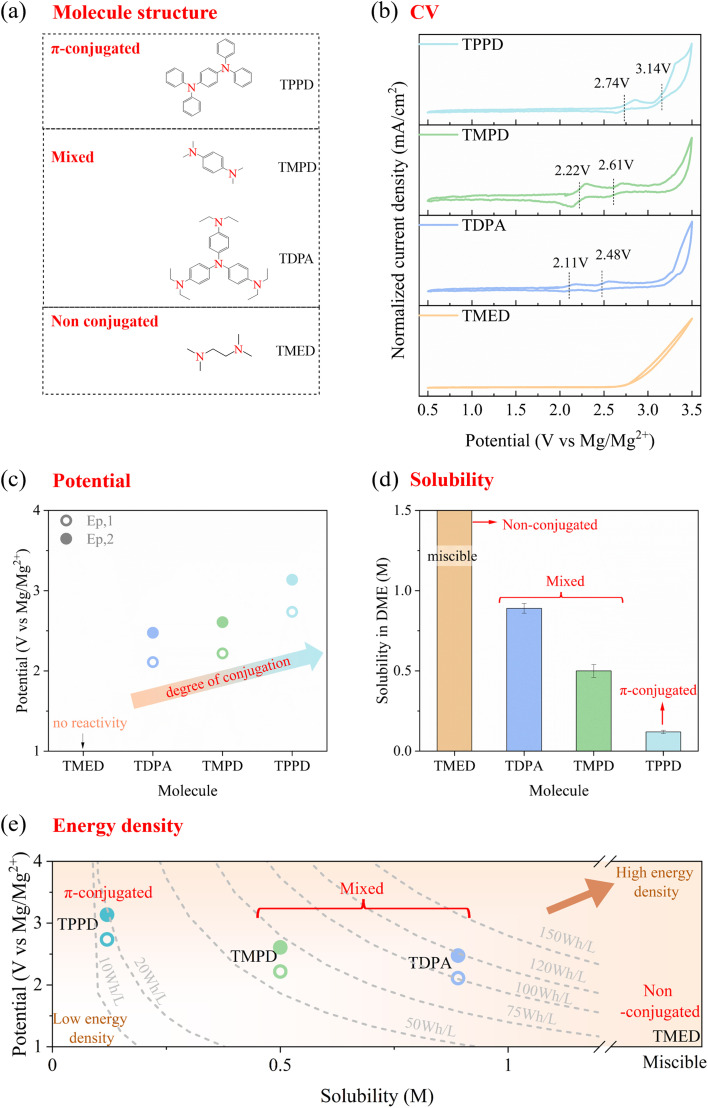
Structural effect on the electrochemical performances of amine molecules. (a) Molecular structure, (b) CV profile, (c) potential, (d) solubility and (e) theoretical energy density of different amine molecules in the Mg electrolyte. The concentration of active materials was set at 1 mM. Hollow circles and solid circles represent the first pair of peaks and the second pair of peaks, respectively.

The amine moiety stood out from the various organic moieties tested. Studies about amines have focused on the *para*-substituted effect on triphenylamine,^30,39–42^ while the effect of π-conjugated and non-conjugated structures has not been explored. Here, four amine molecules (Fig. 3a and Table S3) were chosen to understand how the π-conjugated and/or non-conjugated structures influenced the electrochemical performances and other properties of amine-type molecules. TMED is a non-conjugated amine molecule in which two N atoms are connected to alkyl chains. TPPD is a fully π-conjugated molecule in which two N atoms are connected to phenyl groups. TDPA and TMPD are mixed structures in which two N atoms are both connected with alkyl chains and phenyl groups, simultaneously. Intuition suggests that TPPD would have the highest delocalization owing to its highly conjugated structure. However, for the mixed conjugated systems, the situation was more nuanced owing to the presence of delocalizing phenyl and electron-withdrawing methyl groups. To quantify this arrangement, the average inverse aromatic fluctuation index (FLU^−1^) of these molecules was calculated in their neutral state (see Methods section). A higher FLU^−1^ value corresponds to stronger aromaticity, indicating higher electron delocalization. The degree of conjugation followed the order: (TMED <) TDPA < TMPD < TPPD (Fig. S3), with TMED showing the least conjugation. Although the FLU^−1^ value for TMED was not calculated, it was evident that its conjugation was minimal.

As shown in CV profiles (0.5–3.5 V *vs.* Mg/Mg^2+^) (Fig. 3b), only TMED showed a visible oxidation peak, indicating that molecules with a non-conjugated structure did not have reversible redox reactivity in this condition. The remaining molecules with π-conjugated structures showed two pairs of redox peaks, demonstrating their ability to deliver twice as much capacity compared with one-electron redox couples, such as TEMPO. As their degrees of conjugation increased, their redox potential also increased (Fig. 3c), demonstrating that the electron delocalization in a π-conjugated structure was beneficial to achieve a high potential. The maximum potential was realized by TPPD at 2.74 V *vs.* Mg/Mg^2+^ for the first redox reaction and 3.14 V *vs.* Mg/Mg^2+^ for the second redox reaction. To understand the origins of these experimental trends, the redox potential and highest occupied molecular orbital (HOMO) (Fig. S4) were calculated by DFT from the neutral to charged state. The qualitative trends mirrored the experimental results. These results highlighted the crucial role of conjugation (or electron delocalization) in stabilizing the molecule and decreasing the HOMO energy and increasing the redox potential. A molecule with a highly stabilized oxidation state (lower HOMO energy) requires more energy—or a higher redox potential—to undergo oxidation. Importantly, the effect of conjugation on the redox potential of organic molecules is influenced by their functional groups.^43^ Organic molecules with conjugating backbones exhibit progressively higher redox potentials as more aromatic rings are incorporated, owing to the strengthened inductive effect. This inductive withdrawal of localized electron density increases the redox potential. For the non-conjugating family, the opposite trend is shown. In our study, the amine functional group belonged to the former category because the electron cloud of the lone pair electrons in nitrogen could overlap with and delocalize into the π orbitals of the aromatic ring, resulting in a conjugated structure.^44^

The solubilities of these molecules were tested in DME solutions (Fig. 3d). The non-conjugated molecule, TMED, was miscible with DME, while the fully π-conjugated molecule, TPPD, showed the lowest solubility (∼0.12 M). Molecules with mixed structures exhibit moderate solubilities, ranging from approximately 0.5 to 0.89 M. We compared the HSPs of DME (the solvent), benzene (used as an approximation for the phenyl group in a conjugated structure), methane and ethane (used as an approximation for the alkyl group in a non-conjugated structure) (Table S4). The *δ*_D_ describes dispersion forces and non-polar interactions. *δ*_P_ describes polar forces and dipole–dipole interactions. *δ*_H_ describes hydrogen bonding forces. As the three parameters of the solute and solvent become more similar, the solubility tends to increase. Given that benzene, ethane, and methane are non-polar molecules and have a low ability to form hydrogen bonds, dispersion forces (*δ*_D_) emerged as the primary contributing factor. The Δ*δ*_D_ difference between benzene and DME (3.0) was significantly larger than that between alkanes and DME (1.4 for ethane and 0.1 for methane). As a result, the non-conjugated molecule, TMED, was fully miscible with DME, whereas the fully π-conjugated molecule, TPPD, exhibited the lowest solubility. Other molecules with partial π-conjugation and some alkyl groups displayed moderate solubility in DME.

Theoretical energy densities were calculated for these molecules (Fig. 3e). Molecules with mixed structures showed theoretical energy densities of 50–120 Wh L^−1^, while the theoretical energy densities of non-conjugated molecules and π-conjugated molecules were 0 and ∼10 Wh L^−1^ due to low reversible reactivity or low solubility. Overall, mixed structures offered a balance of optimized potential and solubility. Among all molecules, TDPA demonstrated the most promising performance, achieving a theoretical energy density of ∼120 Wh L^−1^.

**Fig. 4:**
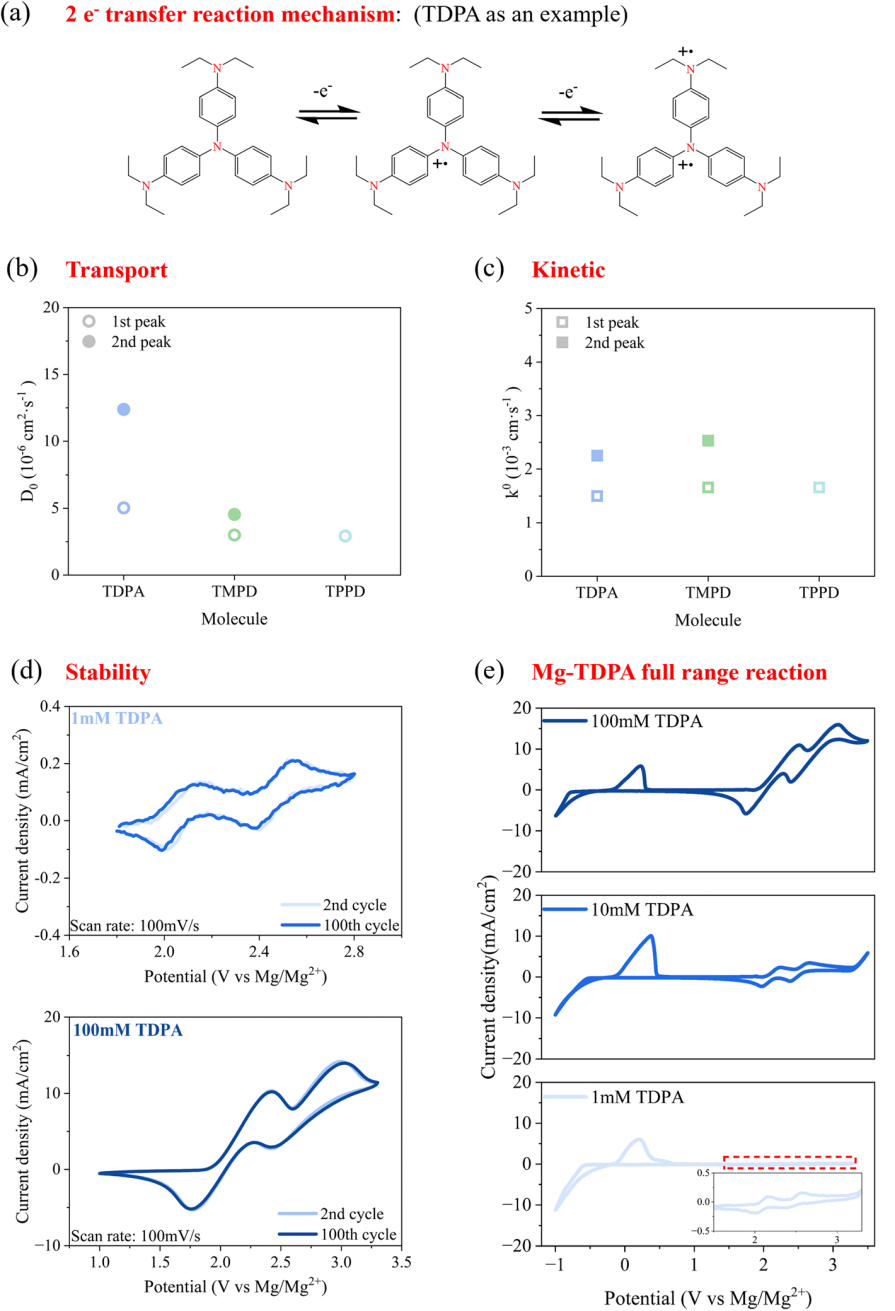
Properties of amine molecules. (a) Reaction mechanism, (b) transport and (c) kinetics. (d) Stability of TDPA with different TDPA concentrations. (e) CV profiles of Mg–TDPA in the full range with different TDPA concentrations.

The reaction mechanism is provided in Fig. 4a using TDPA as an example. It underwent a two-electron transfer reaction,^45^ and two pairs of peaks were obtained for TDPA/TDPA^+^ and TDPA^+^/TDPA^2+^ couples, respectively. The mass transport (Fig. 4b), kinetics (Fig. 4c) and stabilities (Fig. 4d and S5) of these molecules were evaluated by CV at a concentration of 1 mM in the Mg electrolyte. The diffusion coefficient *D*_0_ (Fig. S6a–c) and heterogeneous reduction rate constant *k*^0^ (Fig. S6d–f) were calculated from CV data at different scan rates, according to the reported method.^46^ The observed diffusion coefficient values were in the range of 3–18 × 10^−6^ cm^2^ s^−1^, which is in the same order as those of the most commonly used organic catholyte molecules in flow batteries (Fig. S6g). The reaction rates of these amines were lower than those of organic cathode molecules but higher than those of other common inorganic redox couples such as V^3+^/V^4+^ and Fe^2+^/Fe^3+^.^47^ The CV curves of TDPA (Fig. 4d) and TMPD (Fig. S5a) at the second and 100th cycles at the scan rate of 100 mV s^−1^ almost completely overlapped with each other, demonstrating the high stabilities of these amine redox couples. It is worth noting that these curves exhibited fluctuations due to the low concentration of active material, which resulted in a decreased signal-to-noise ratio. To further investigate the effect of concentration, we tested TDPA catholyte (100 mM) under the same conditions, as shown in Fig. 4d. With increased concentration, CV displayed smoother profiles. Although the redox potential experienced a slight shift, the stability remained unchanged. For the fully π-conjugated molecule, TPPD, its second redox peak (Fig. S5b) faded during cycling, which may have been caused by the electrolyte decomposition beyond 3.00 V *vs.* Mg/Mg^2+^ (Fig. S5d). When the upper-limit scan range was set to 3.00 V *vs.* Mg/Mg^2+^, TPPD showed completely overlapping profiles during 100 cycles (Fig. S5c). The full-range scan, covering the anode (Mg ↔ Mg^2+^) and cathode reactions (*e.g.*, TDPA ↔ TDPA^2+^), was conducted at TDPA concentrations of 1 mM, 10 mM, and 100 mM (Fig. 4e). As the TDPA concentration increased, the current response of the TDPA ↔ TDPA^2+^ reaction also increased, while the redox potential remained relatively consistent, with only slight shifts observed. Meanwhile, the current response and redox potential of the Mg ↔ Mg^2+^ reaction were close across different TDPA concentrations. These results indicated that the TDPA and Mg reactions did not interfere with each other. In general, amine molecules showed high diffusivities, moderate kinetics and high stabilities in Mg electrolyte systems, implying that they are favorable for low-polarization, high-efficiency and high-stability Mg RFBs.

### Demonstration of a Mg flow battery

**Fig. 5:**
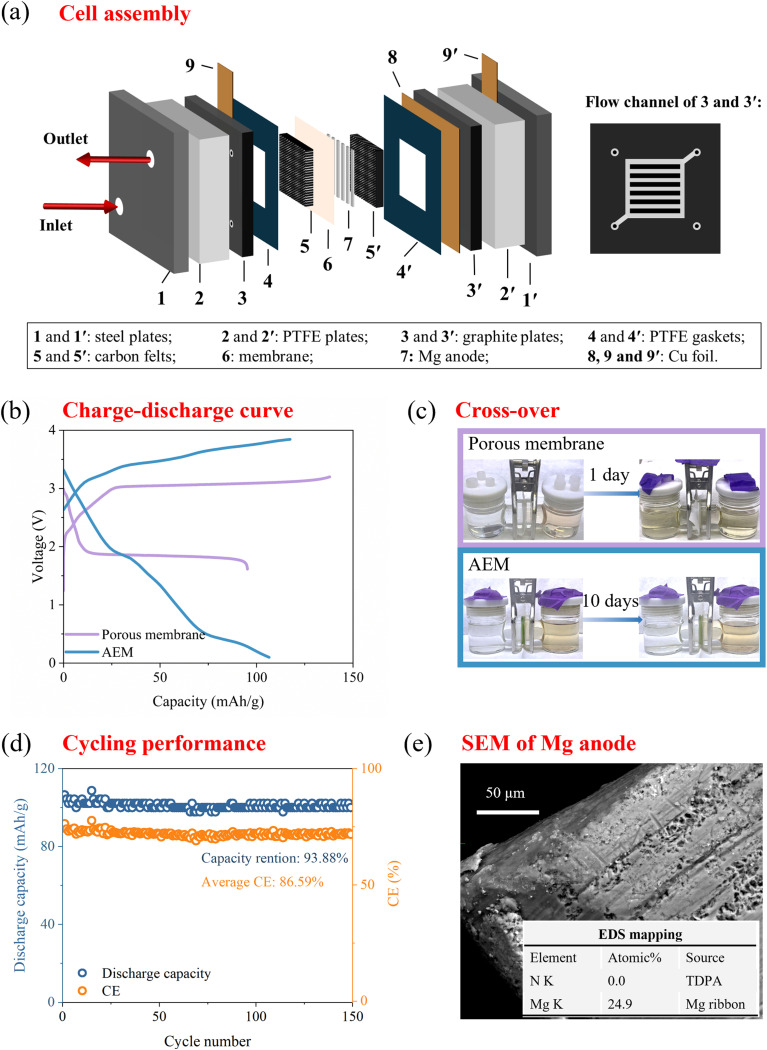
Demonstration of a Mg flow battery using TDPA as an example. (a) Cell assembly. (b) Charge–discharge curve. (c) Crossover of TDPA through a porous membrane and AEM. Left side of H-cell: pure DME; right side of H-cell: TDPA (1 mM) in DME. (d) Cycling performance at 10 mA cm^−2^ (e) SEM and EDX of the Mg ribbon anode after cycling.

To confirm its exceptional performance for practical applications, we further examined the electrochemical performance of TDPA at the device level in a flow cell. The cell construction is shown in Fig. 5a, and experimental details can be found in the Methods section. The typical CV curves of the electrolyte and TDPA are shown in Fig. 4e, which demonstrated the feasibility of a flow cell with a high voltage of ∼2.70 V. RFBs using Mg ribbons as the anode, a porous membrane as the separator, and TDPA as the redox-active catholyte molecule were assembled and discharged/charged galvanostatically. As shown in Fig. 5b, the Mg–TDPA cell could deliver an average voltage of 2.50 V, a specific discharge capacity of 95.3 mAh g^−1^ (calculated by the weight of the TDPA molecule) with a first cycle coulombic efficiency (1st CE) of 69.07%. The low CE was caused by severe crossover of TDPA molecules through the porous membrane, which did not pose a barrier for amine molecules to diffuse through. As evidenced in Fig. 5c, the porous membrane showed significant crossover within one day, as indicated by discoloration. Amine molecules belong to p-type molecules, so their states are neutral at the discharged state and positively charged at the charged state. To block the crossover of neutral or positively charged TDPA, an anion exchange membrane (AEM) was used to replace the porous membrane, and the crossover test showed it limited crossover even after 10 days, demonstrating its superior barrier properties. Such an Mg–TDPA cell using an AEM membrane could deliver a specific discharge capacity of 106.5 mAh g^−1^ (corresponding to a volumetric capacity of 49 mAh L^−1^), a 1st CE of 90.74%, and achieve 93.88% capacity retention after 150 cycles (Fig. 5d). After disassembling the cell, the cycled Mg ribbon was examined by scanning electron microscopy (SEM) and energy dispersive spectroscopy (EDS). As shown in Fig. 5e, the Mg ribbon surface had obvious holes, indicating deposition–dissolution of Mg metal. EDX mapping also showed signals for Mg. The signal for N in the TDPA molecule was missing in EDX, demonstrating that AEM inhibited the crossover of TDPA. The excellent cycling stability was consistent with the molecular stability demonstrated in Fig. 2d, which revealed the great potential of amine molecules to construct long-life Mg RFBs.

The increased overpotential between charge and discharge, along with the low voltage efficiency and energy efficiency (Fig. S7), was a result of the poor ionic conductivity of the AEM in such non-aqueous electrolytes. Meanwhile, the incomplete CE and slight chemical change of TDPA catholyte after cycling (Fig. S8) may have been caused by membrane swelling. When using an AEM membrane, the cell exhibited smooth flow when only pumping the Mg electrolyte. However, upon addition of 0.1 M TDPA, the membrane swelled, causing flow disruption at the cell outlet. This phenomenon indicated that amine molecules interacted with the AEM, leading to swelling. Notably, this swelling effect diminished as the concentration decreased. Therefore, flow cell tests were conducted with low active material concentration, and performance at higher concentrations was not evaluated. It should be noted that the focus of this work was on the design of the organic molecule catholyte for Mg RFBs, whereas the design of highly conductive and highly stable ion-selective membranes for non-aqueous flow batteries, although important, was beyond the scope of the current study.

### Electrolyte influence of the amine-based catholyte in RFBs

The electrolyte was prepared by dissolving 0.5 M LiTFSI, 0.25 M Mg powder, and 0.5 M MgCl_2_ in DME. The active species was [Mg_2_(μ-Cl)_2_]^2+^ cation complex, which is highly active for reversible Mg-based reactions.^48^ This complex was formulated in DME through dehalodimerization of non-nucleophilic MgCl_2_ by reacting with LiTFSI. The latter was chosen over Mg(TFSI)_2_ due to its higher purity and the absence of any interference from Mg^2+^ activity.^49^ Mg powder was added to remove residual moisture. The effects of electrolyte solvent and salt composition on amine molecules are unveiled in this section. We primarily focused on exploring organic cathode molecules that were compatible with the Mg anode, so the impact of different electrolytes on the redox properties of Mg anode was not explored in detail.

**Fig. 6:**
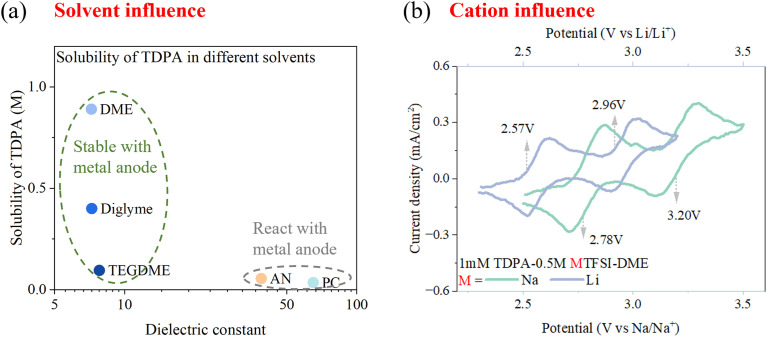
Electrolyte influence. (a) Solubility of TDPA in different solvents with different polarities. (b) CV profiles of TDPA–DME (1 mM) using different cations.

The catholyte in this work employed a glyme solvent, known for its weak polarity. As mentioned briefly in the introduction, high-polarity solvents are used in most non-aqueous RFBs to achieve the high ionic conductivity of the electrolytes. According to the CV profiles (Fig. S9a), the choice of solvent did not affect the redox potential of amine-based redox-active molecules. The only difference was that the AN system could achieve a higher current density than the DME system due to the high conductivity of the AN-based supporting electrolyte (Fig. S9b). To improve the kinetics of TDPA in DME, the flow rate in Mg RFBs could be increased, making conductivity a less critical concern. More importantly, there were two key challenges to using AN/propylene carbonate (PC)-based supporting electrolytes in Mg-amine RFBs (Fig. 6a): (1) low solubility of amine molecules (Table S5) and (2) high reactivity with the metal anode. The solubility of TDPA decreased with an increase in solvent polarity. For example, the solubility of TDPA in DME was 16-times and 25-times higher than that in AN and PC, which are the most used solvents in non-aqueous RFBs. As shown in Table S4, the phenyl and alkyl groups in amines had a *δ*_P_ of 0, DME had a moderate *δ*_P_ of 6, while AN and PC had *δ*_P_ of 18. This large difference led to the extremely low solubility of amines in highly polar solvents. Similar solubility differences could also be observed for TPPD (Table S6). Moreover, these high-polarity solvents could react with metal anodes and compromise the stability of Mg RFBs. Hence, DME was identified as the best solvent due to its highest solubility and high compatibility with the metal anode, challenging the prevailing view that high-polarity solvents are essential in non-aqueous RFBs.

Additionally, it is worth noting that the amine-based cathode molecule was a p-type active material. When the amines were oxidized, the positively charged molecules could interact with anions *via* electrostatic forces, and this binding may influence the peak potential. TFSI^−^ and ClO_4_^−^ were selected to investigate the bulk anion influence on TDPA performance, and their CV profiles showed similar redox peaks (Fig. S10a). FTIR spectroscopy was conducted to study the chemical bonding environment of TDPA (Fig. S10b). Compared with pure DME solvent, the profile of TDPA–DME showed a characteristic peak at 1507.121 cm^−1^, indicating the presence of TDPA. When 0.5 M TFSI^−^ or ClO_4_^−^ was added to the TDPA–DME solution, there was no change in peak position, which meant that the chemical environment of TDPA was unchanged by the anions. Based on the combined electrochemistry and spectroscopy results, we inferred that TFSI^−^ or ClO_4_^−^ were only weakly associated with the TDPA molecule and did not significantly influence its redox potential. These amine-based catholytes were also applicable to Na and Li RFBs (Fig. 6b) due to the compatibility of ether with the metal anode. In Li-based and Na-based electrolytes, the TDPA catholyte also enabled two-electron-transfer reactions (2.57 and 2.96 V *vs.* Li/Li^+^, 2.78 and 3.20 V *vs.* Na/Na^+^).

## Conclusions

Nonaqueous Mg RFBs are attractive for low-cost, high-energy-density and long-cycle-life stationary energy storage applications. A comprehensive experimental and computational study was conducted to examine organic molecules of different redox moieties and conjugated structures for Mg RFBs. Amine molecules were identified as the most suitable catholyte molecule for Mg RFB due to their high redox potential, excellent reversibility, and superior stability. The critical influence of π-conjugated and non-conjugated structures of amines on their thermodynamics, solubility, kinetics, transport properties, and stability in Mg RFB was systematically studied, highlighting the contribution of this work compared with earlier reports on an amine-based flow battery that primarily focused on the *para*-substituted effects of triphenylamine on redox potential and solubility.^30,39–42^

A key finding of this work was that the redox potential of the amine molecule scaled with the degree of conjugation; a higher degree of conjugation resulted in a higher redox potential of the catholyte. TDPA with mixed π-conjugated and non-conjugated structures showed the best performance among all the studied amine molecules due to a balance of optimized potential and solubility. When paired with an Mg anode, a flow battery employing TDPA could achieve energy densities as high as ∼120 Wh L^−1^ for a concentration of 0.89 M and the observed redox potential of 2.70 V *vs.* Mg/Mg^2+^. An Mg-amine redox flow battery using TDPA as the catholyte molecule was demonstrated, which delivered a voltage of 2.50 V, a specific discharge capacity of 106.5 mAh g^−1^, an average CE of 86.59%, and achieved 93.88% capacity retention after 150 cycles. This excellent performance was attributed to the mixed π-conjugated and non-conjugated structures in TDPA, as well as its high stability and high solubility in the ether-based supporting electrolyte. The crossover of the amine molecule could be significantly suppressed by replacing a porous membrane with an AEM at the expense of increasing resistance, overpotential and swelling. This phenomenon highlights the need of membrane research to fully materialize the potential of Mg RFBs, which is beyond the scope of this work.^50^ In addition to non-aqueous Mg RFBs, this class of redox-active molecules is also applicable in various hybrid flow cells such as Li RFBs and Na RFBs, expanding the applications and opportunities of amine-based catholytes in flow batteries.

From the perspective of molecular design, incorporating more alkyl/glycol chains and phenyl groups into amine-based organic molecules is a promising strategy to enhance the electrochemical performance of amine-based molecules and address challenges in cell construction. This strategy has four advantages: first, these organics feature an amine core, as demonstrated in this work, enabling a two-electron transfer reaction, high voltage, and improved stability. Second, their long alkyl chains and/or polyethylene glycol chains (non-conjugated structures) enhance solubility in ether-based solvents. Third, incorporation of more phenyl groups (π-conjugated structures) increases stability and redox potential. Lastly, from a cell-construction perspective, incorporating more alkyl/glycol chains and phenyl groups increases molecular size, thereby minimizing crossover through the porous membrane and eliminating the need for high resistance, swelling-prone ion-exchange membranes. Therefore, this work paves the way for rational structural design of organic molecules for Mg-based redox flow batteries.

## Methods

### Catholyte preparation

All organic molecules used in Fig. 2 are listed in Table S1, including their abbreviations, molecular weights, purities and vendors. The four selected anime-based molecules are listed in Table S3 with their properties and vendors. All organic molecules were used as received without further processing. Preparation of the catholytes was conducted by dissolving the cathode molecules in the prepared electrolyte at room temperature without stirring. The supporting electrolytes for Mg RFBs were prepared as follows. Briefly, 0.5 M LiTFSI, 0.25 M Mg powder and 0.5 M MgCl_2_ were added to DME, and the mixture was stirred for 6 h at 60 °C, and then the solutions were filtered. The supporting electrolytes for Na RFBs and Li RFBs were prepared by dissolving 0.5 M of the desired salt in the desired solvent. All operations were conducted in a N_2_ (99.999%)-filled glove box (O_2_ < 1 ppm and H_2_O < 1 ppm).

Magnesium dichloride (MgCl_2_, 99.99%), Mg powder (50 mesh, >99%), and DME (99.5%), diglyme (anhydrous, 99.5%), tetraethylene glycol dimethyl ether (TEGDME, >98%), propylene carbonate (PC, 99.7%), and AN (99.8%) were purchased from MilliporeSigma (formerly Sigma-Aldrich). Other salts used in this work were sodium perchlorate (NaClO_4_, 98%, Thermo Scientific), sodium bis(trifluoromethanesulfonyl)imide (NaTFSI, 99.5%, Solvionic) and lithium bis(trifluoromethanesulfonyl)imide (LiTFSI, 99.5%, Gotion).

### Materials characterization

Fourier-transform infrared (FTIR) spectroscopy was done using the Nicolet iS50 FTIR spectrometer with a diamond ATR crystal, on which the electrolyte was placed directly on the windows testing holders during the test. The crossover test was done using a two-compartment glass diffusion cell. One compartment was filled with pure DME, and the other was filled with 1 mM TDPA–DME solution. The tested membrane acted as the separator. SEM and EDS were done with an FEI Quanta 600 FEG system. Before analysis, samples were washed with DME solvent and dried under a vacuum.

### Electrochemistry

CV was undertaken with an interface 1010 electrochemical workstation from Gamry Instruments. For the CV of the catholyte, a glass carbon disk (diameter: 3 mm) was used as the working electrode, and two polished Mg foils (or Na, Li disks for the Na and Li system) were used as the counter electrode and the reference electrode, respectively. For a catholyte using AN or PC as the solvent, Pt was used as the counter electrode and Ag/Ag^+^ was employed as the reference electrode. The Ag/Ag^+^ electrode was filled with 0.01 M AgBF_4_ and 0.5 M TBAPF_6_ in AN.

Galvanostatic discharge–charge measurements were performed on a multi-channel battery tester made by Landt Instruments. The cell construction can be found in Fig. 5a. A porous membrane (pore size: 0.034 μm; Shenzhen Jialiye Technology) and AEM (Fumasep FAS-30, Fuel Cell Store) were used as separators. In a typical test, the volume of the catholyte was 10 mL, and the battery was charged and discharged galvanostatically at a current density of 10 mA cm^−2^ at room temperature. For the cell using a porous membrane, we set the cutoff voltage window from 1.6 V to 3.2 V for the TDPA redox reaction. For the cell using the AEM, we set the cutoff voltage window from 0.1 V to 3.8 V. A larger voltage window was used to compensate for the significant overpotential caused by the AEM.

### Calculation for the aromatic fluctuation index

The inverse aromatic fluctuation index (FLU^−1^) is used to quantify the delocalization index of the benzene rings in the catholyte molecule. FLU^51^ is defined as:1
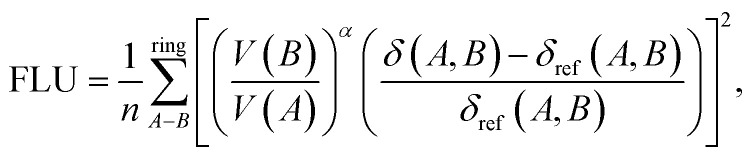
2
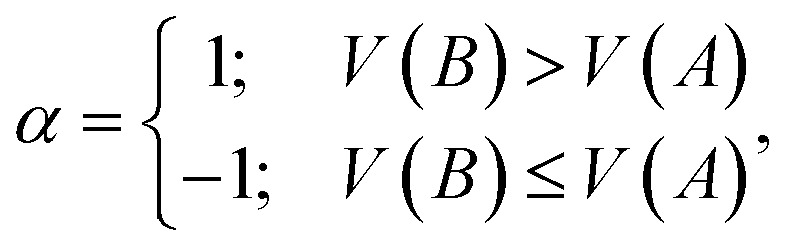
3
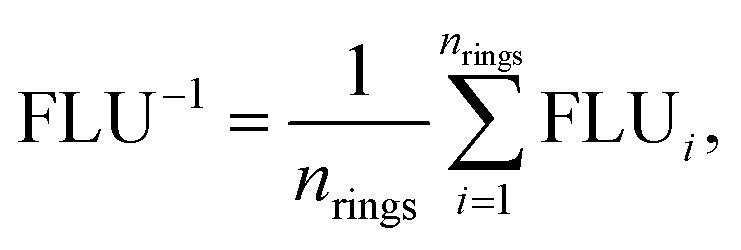
where *A* and *B* denote atoms in the ring, *n*_rings_ represents the number of phenyl rings, *n* represents the number of carbon atoms in each phenyl ring, and *δ*(*A*,*B*) and *δ*_ref_ represent the ring delocalization and reference delocalization indices, respectively. FLU was computed for each phenyl ring and FLU^−1^ was calculated as an average across all rings. Multiwfn^52^ software was used to compute the FLU values for all molecules in their uncharged state.

### Density functional theory calculations

Density functional theory (DFT) calculations were used to compute the redox potential of the different catholytes in solution. DFT calculations were performed with the Gaussian 16 package with the B3LYP/6-31+G(d,f) basis function. Solvent effects were included implicitly using the polarizable continuum model (PCM). Experiments were performed in glyme. The Gaussian 16 package did not have the parameters corresponding to this solvent. Among the available solvents in the Gaussian 16 package, we chose diethyl ether, whose structure and dielectric constant (4.20) are similar to those of glyme (7.20).

## HOMO–LUMO

The energy difference between the highest occupied molecular orbital (HOMO) and lowest unoccupied molecular orbital (LUMO) was computed for the uncharged state in a solvent. Multiwfn software was used to obtain HOMO and LUMO energies.

## Author contributions

Yunan Qin: conceptualization, data curation, data analyses, investigation, methodology, validation, visualization, and writing (original draft). Vaidyanathan Sethuraman: data curation, data analyses, methodology, and software. Seong-Gyu Choi: investigation and data analyses. Richard Gonzalez: investigation and data analyses. Chengxiang Chen: investigation and writing (review and editing). Lei Cheng: project administration, resources, and supervision. Chao Luo: conceptualization, funding acquisition, project administration, supervision, and writing (review and editing). Tao Gao: conceptualization, funding acquisition, project administration, resources, supervision, and writing (review and editing).

## Conflicts of interest

There is no conflict of interest to declare.

## Supplementary Material

SC-OLF-D5SC04532K-s001

## Data Availability

The data supporting the conclusions reached from our study have been included as part of the SI. See DOI: https://doi.org/10.1039/d5sc04532k.
