# Supplementary material for: Conjugation effect of amine molecules in non-aqueous Mg redox flow batteries
Source: Chem Sci. 2025 Jul 31;16(35):16205–17. doi: 10.1039/d5sc04532k (PMC12368905; doi:10.1039/d5sc04532k)
Supplement: SC-016-D5SC04532K-s001 [file SC-016-D5SC04532K-s001.pdf]

Supplementary Information (SI) for Chemical Science.
This journal is © The Royal Society of Chemistry 2025

Supporting information

Conjugation effect of amine molecules in non-aqueous Mg redox flow batteries

Yunan Qin [a], Vaidyanathan Sethuraman [b], Seong-Gyu Choi [a], Richard Gonzalez [a],

Chengxiang Chen [c], Lei Cheng [b], Chao Luo [c]*, Tao Gao [a]*

[a] Department of Chemical Engineering, University of Utah, Salt Lake City, UT, USA

[b] Chemical Sciences Division, Oak Ridge National Laboratory, Oak Ridge, TN, USA

[c] Department of Chemical, Environmental and Materials Engineering, University of Miami, Coral Gables, FL, USA

*Corresponding: taogao@chemeng.utah.edu; cxl1763@miami.edu.

Content

Tables

Table S1. Abbreviations, molecular weights, purities and vendors of selected organic molecules with different redox moieties.

Table S2. Summary of the redox potentials of selected organic molecules with various redox-active moieties.

Table S3. Abbreviations, molecular weights, purities and vendors of selected amines.

Table S4. The Hansen Solubility Parameters (HSP) of solutes and solvents.

Table S5. Solubilities of TDPA in common organic solvents.

Table S6. Solubilities of different amines in DME and AN.

Figures

Figure S1. Reaction mechanism of selected organic molecules with different redox moieties.

Figure S2. CV profiles (2nd, 100th and 300th cycle) of selected organic molecules with different redox moieties.

Figure S3. Calculated average of the inverse of aromatic fluctuation index.

Figure S4. Calculated oxidation potential and HOMO.

Figure S5. Transport and kinetics of amine molecules.

Figure S6. Stabilities of different amine molecules.

Figure S7. Voltage efficiency (VE) and energy efficiency (EE) of the Mg-amine flow cell using anion exchange membrane.

Figure S8. FTIR of catholyte before and after test.

Figure S9. CV profiles of 1 mM TDPA-0.5 M NaTFSI dissolved in different solvents.

Figure S10. FTIR of different solutions. The concentration of TDPA is 1 mM in these solutions.

Computational methods

Tables

Table S1. Abbreviations, molecular weights, purities and vendors of selected organic molecules with different redox moieties.

Name	Abbr.	Molecular Weight (g/mol)	Purity	Source
Azobenzene	AB	182.23	98%	Thermo Scientific Chemicals
Anthraquinone	AQ	208.21	97%	Sigma-Aldrich
Tetrachloro-1,4-benzoquinone	TCBQ	245.88	99%	Sigma-Aldrich
(2,2,6,6-Tetramethylpiperidin-1-yl)oxyl	TEMPO	156.25	98%	Sigma-Aldrich
N,N,N′,N′-Tetraphenyl-1,4-phenylenediamine	TPPD	416.54	≥98%	TCI America™
1,10-Phenanthroline	Phen	180.21	≥99%	Sigma-Aldrich
Diphenyl sulfide	DPS	186.27	98%	Sigma-Aldrich
1,4-Di-tert-butylbenzene	DTBB	190.32	98%	Sigma-Aldrich

Table S2. Summary of the redox potentials of selected organic molecules with various redox-active moieties.

**Molecule**	**Type**	E_{pc} **(V vs Mg/Mg^{2+})**	E_{pa} **(V vs Mg/Mg^{2+})**	ΔE **(V)**
AB	*n*	0.50	1.28	0.78
AQ	*n*	1.26	1.84	0.58
TCBQ	*n*	1.89	2.22	0.33
TPPD	*p*	2.56	2.80	0.24
TEMPO	*p*	2.29	2.99	0.70
Phen	*p*	2.72	3.50	0.78
DPS	*p*	2.76	3.50	0.74
DTBB	*p*	2.81	3.50	0.69

Table S3. Abbreviations, molecular weights, purities and vendors of selected amines.

Name	Abbr.	Molecular Weight (g/mol)	Purity	Source
N,N,N′,N′-Tetramethylethylenediamine	TMED	116.24	≥97%	Fisher BioReagents
Tris[4-(diethylamino)phenyl]amine	TDPA	458.68	99%	Sigma-Aldrich
N,N,N',N'-Tetramethyl-p-phenylenediamine	TMPD	164.25	≥98%	Thermo Scientific Chemicals
N,N,N′,N′-Tetraphenyl-1,4-phenylenediamine	TPPD	412.54	≥98%	TCI America™

Table S4. The Hansen Solubility Parameters (HSP) of solutes and solvents. The parameters are from https://www.stevenabbott.co.uk/practical-solubility/hspintro.php.

Name	Classification	δ_D	δ_P	δ_H
Benzene	an approximation for the phenyl group in conjugation structure	18.4	0	2
Methane	an approximation for the alkyl group in non-conjugated structure	14	0	0
Ethane	an approximation for the alkyl group in non-conjugated structure	15.5	0	0
DME	Solvent	15.4	6	6
Diglyme	Solvent	15.7	6.1	6.5
AN	Solvent	15.3	18	6.1
PC	Solvent	20	18	4.1

Table S5. Solubilities of TDPA in common organic solvents.

Solvent	Abbreviation	Dielectric constant	Solubility of TDPA (mol/l)
Dimethoxyethane	DME	7.20	0.86-0.92
Diglyme	Diglyme	7.23	0.33-0.47
Tetraethylene glycol dimethyl ether	TEGDME	7.79	0.09-0.10
Acetonitrile	AN	38.80	0.05-0.06
Propylene carbonate	PC	64.90	0.03-0.04

Table S6. Solubilities of different amines in DME and AN.

Molecule	Solubility in DME (mol/l)	Solubility in AN (mol/l)
TMED	miscible	miscible
TDPA	0.86-0.92	0.05-0.06
TMPD	0.46-0.54	0.46-0.54
TPPD	0.10-0.13	<0.02

Figures

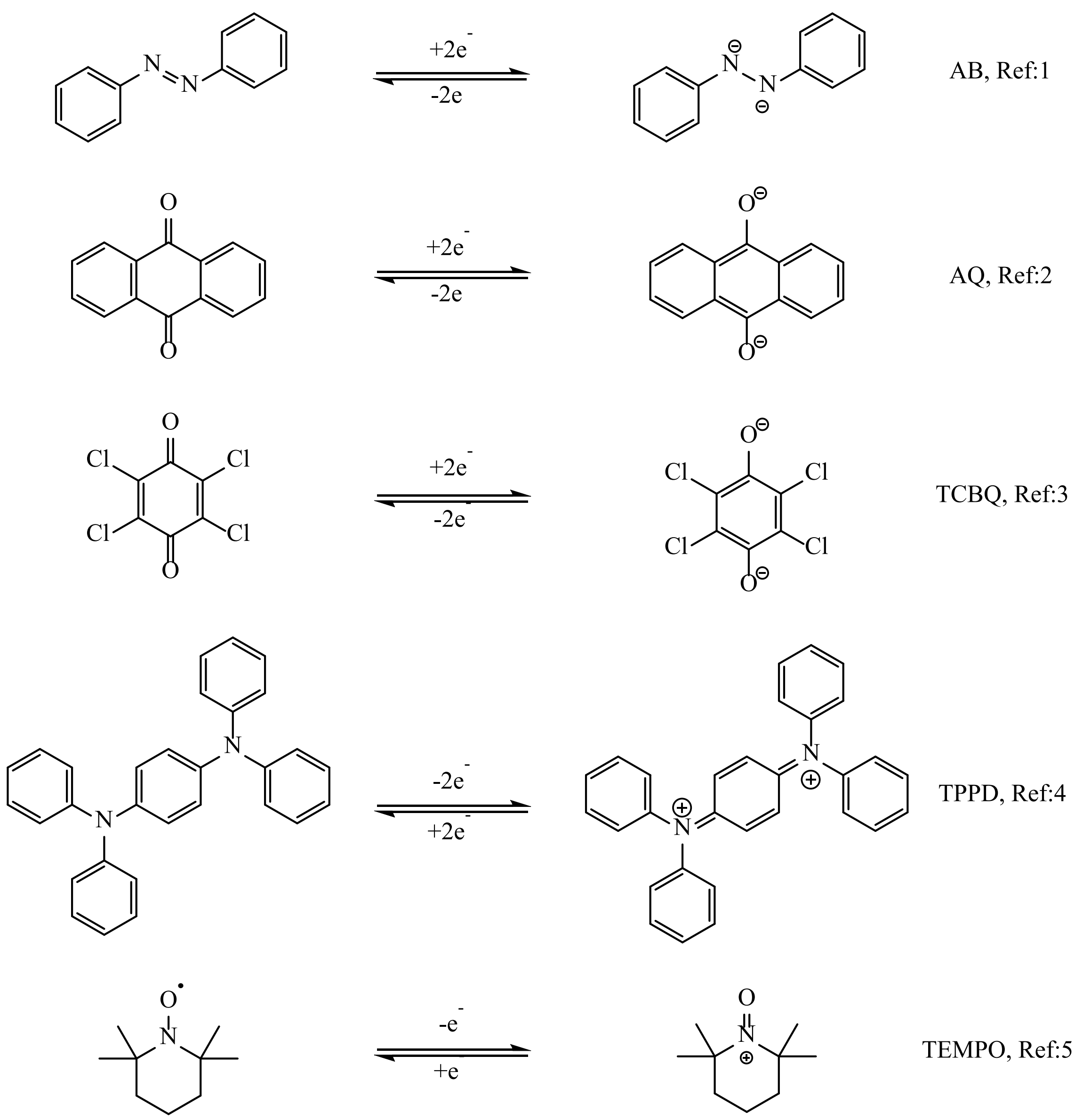

Figure S1. Reaction mechanism of selected organic molecules with different redox moieties.[1,2,3,4,5]

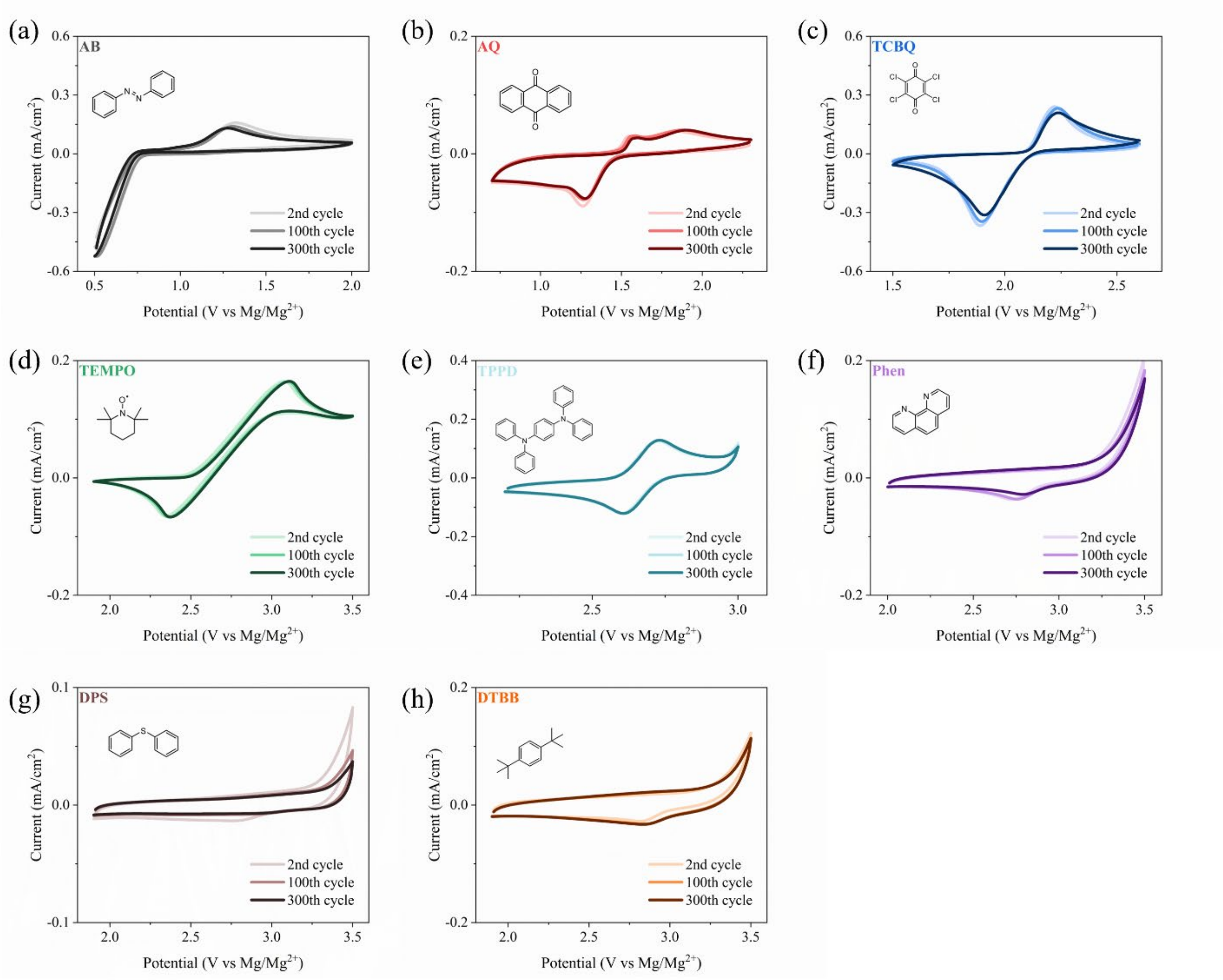

Figure S2. CV profiles (2nd, 100th and 300th cycles) of selected organic molecules with different redox moieties. Scan rate: 100 mV/s.

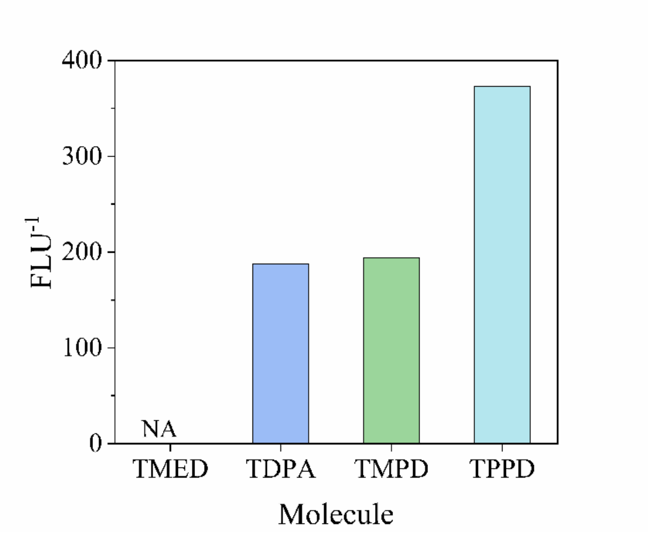

Figure S3. Average of the inverse of aromatic fluctuation index.

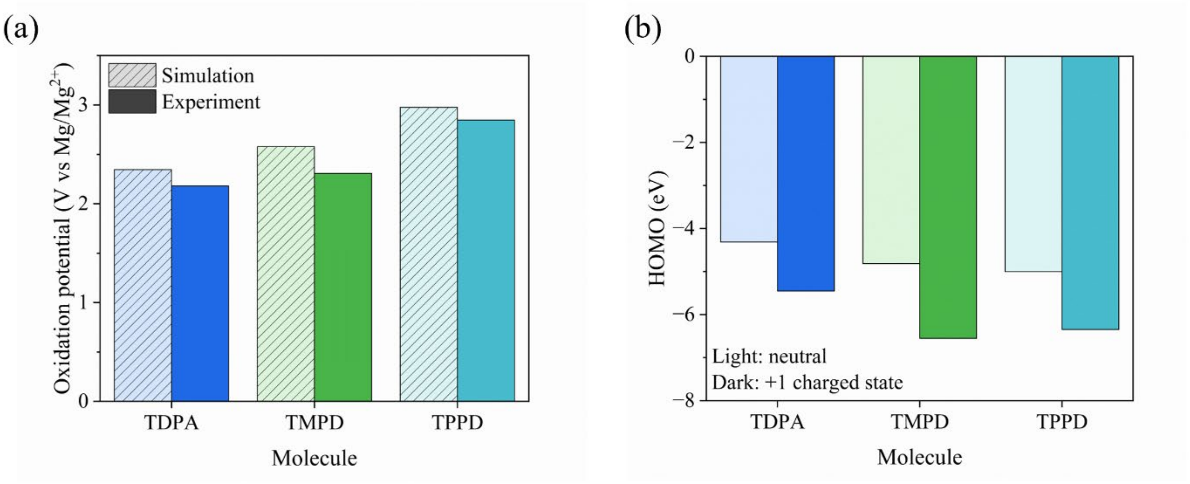

Figure S4. (a) Simulated and experimental oxidation potential from neutral to +1 state. Oxidation potentials are calculated using Eq. (S1). (b) HOMO levels of neutral and +1 states.

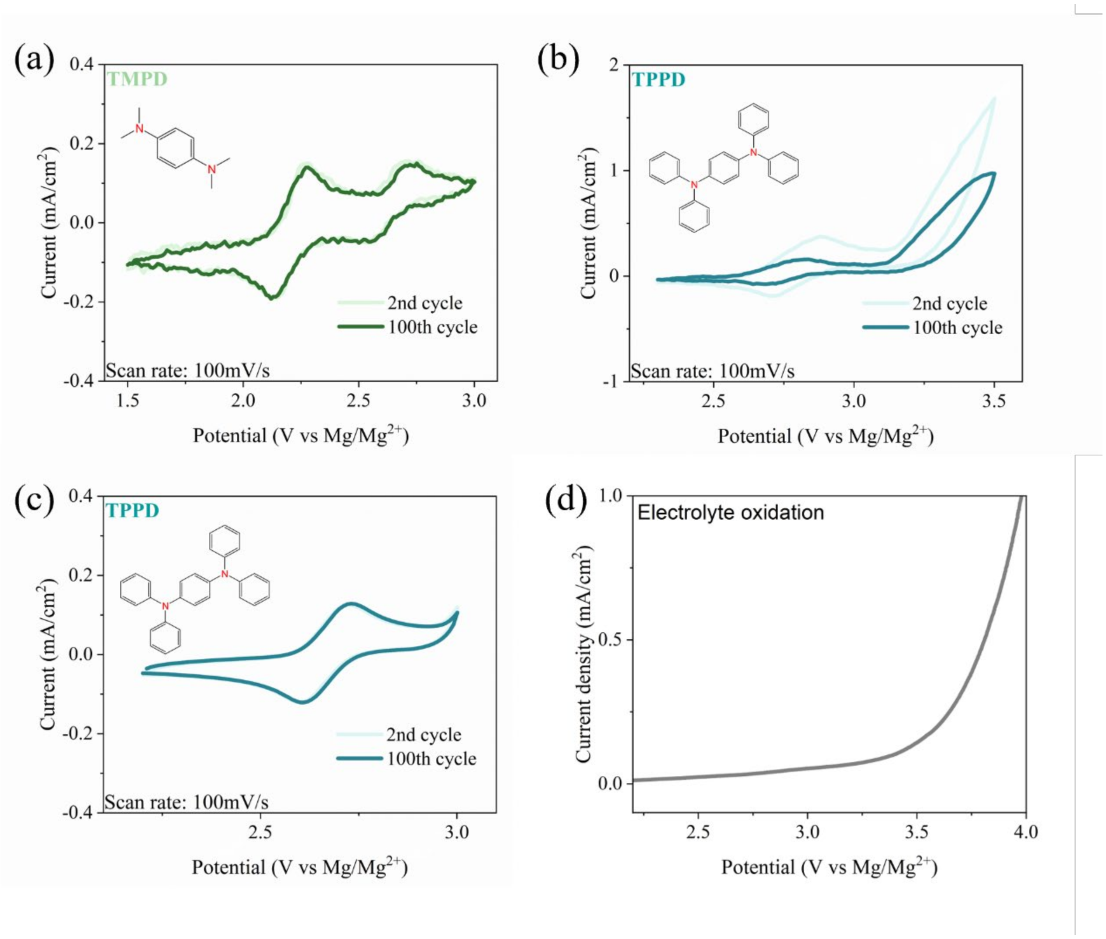

Figure S5. (a-c) Stabilities of different amine molecules. (d) Linear sweep voltammetry (LSV) of Mg-based electrolyte.

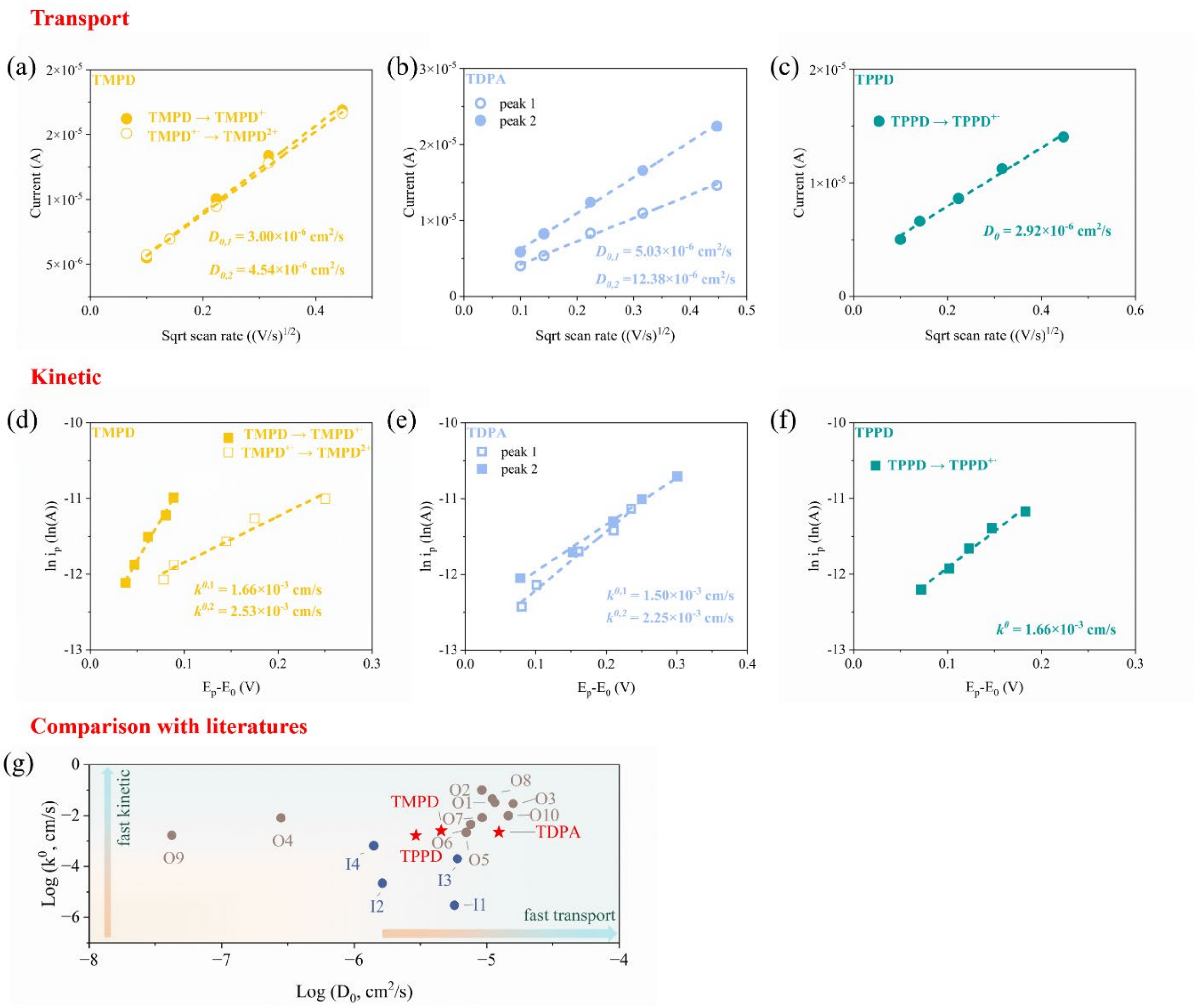

Figure S6. (a-c) Transports and (d-f) kinetics of amine molecules. (g) Comparison of amine molecules with common organic (O) and inorganic (I) redoxmers in literature.

Inorganic(I)			Organic (O)		
Abbreviation	Redoxmer	Reference	Abbreviation	Redoxmer	Reference
I1	VO2+	6	O1	3MTPA	7
I2	Fe2+	6	O2	TEMPO	8
I3	Cr3+	6	O3	DMPZ	9
I4	V(acac)3	10	O4	isoV	11
			O5	CP	12
			O6	AB	13
			O7	TCNE	14

	O8	NMPI	[15]
	O9	OFN	[16]
	O10	DBMMB	[17]

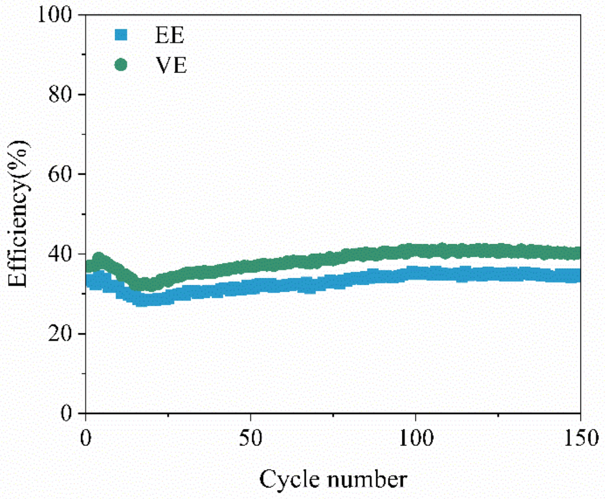

Figure S7. Voltage efficiency (VE) and energy efficiency (EE) of the Mg-amine flow cell using anion exchange membrane.

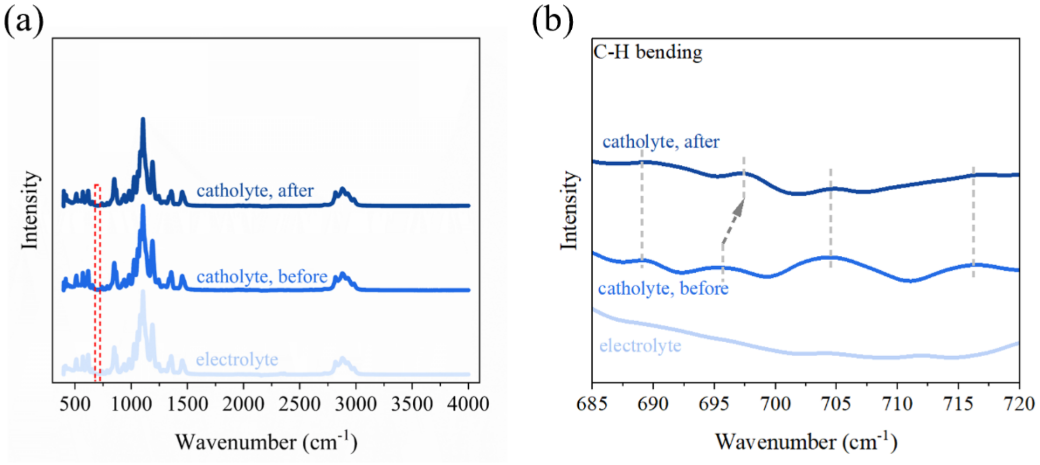

Figure S8. FTIR of catholyte before and after test. (a) Full range and (b) Zoom-in of the C-H bending region.

Discussion: the supporting electrolyte and catholyte before and after test were examined by Fourier Transform Infrared Spectroscopy (FTIR) spectra (**Figure S6**). Compared with the supporting electrolyte, catholyte before test show multiple peaks around 685-720 cm^{-1} attributed to C-H bending of TDPA's phenyl group. After cycling, peak at 696 cm^{-1} shows a little shift to higher wavenumbers, indicating structure changes of TDPA during cycling. Together with an average CE of 86.59%, these results indicate that Mg-TDPA still shows somewhat irreversibility during flow cell cycling, which may be caused by TDPA's insufficient kinetics and the side reaction such as dimerization[18] of TDPA at oxidized states.

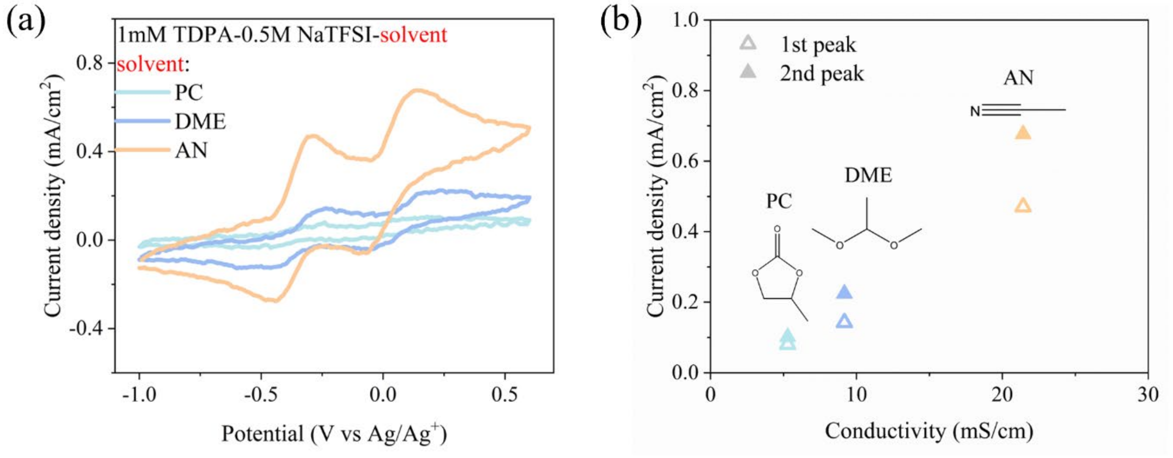

Figure S9. (a) CV profiles of 1 mM TDPA-0.5 M NaTFSI dissolved in different solvents. (b) Correlation of solvent polarity with the peak current density.

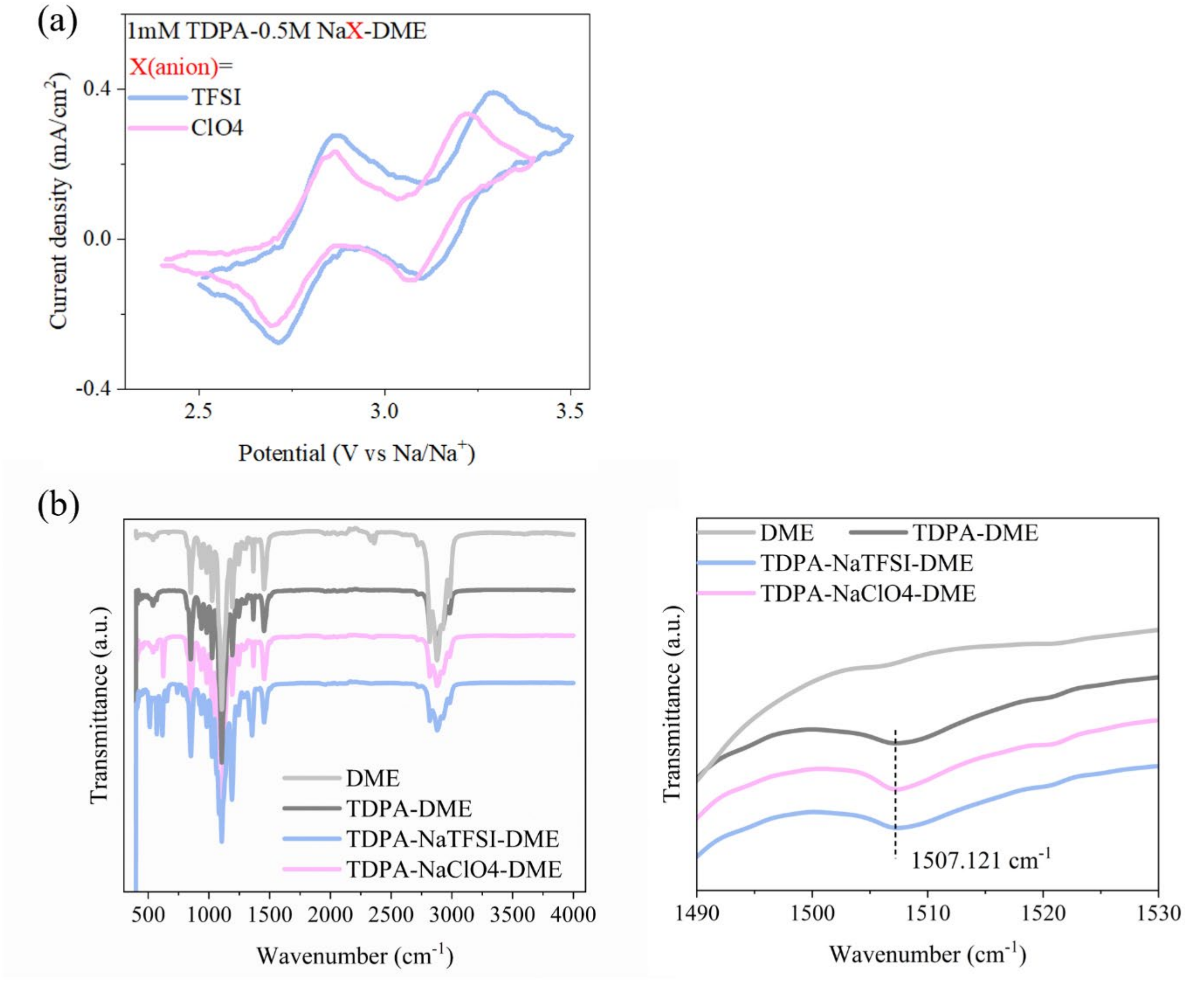

Figure S10. (a) CV profiles of 1mM TDPA-DME using different anions. (b) FTIR of different solutions (Left: full range; Right: zoom-in range).

Computation

Quantification measures

Redox Potential

We calculate the redox potential using the following relation,

$$E^{ox} = \frac{\Delta G_s^{ox}}{nF} - E_{ref}, \dots \text{(S1)}$$

where E^{ox} corresponds to the oxidation potential, ΔG_s^{ox} corresponds to the change in Gibbs free energy of arising from the oxidation of the molecule in solvent, n corresponds to the number of electrons transferred, and F corresponds to the Faraday's constant (96500 C/mol). The constant E_{ref} denotes the difference in the redox potential between Mg^{2+}/Mg (-2.37 V) and the standard hydrogen electrode (-4.44 V) and the yielding a value of 2.07 V. The sign for ΔG is positive since we are considering oxidation here.

HOMO-LUMO

The energy difference between the highest occupied molecular orbital (HOMO) and lowest unoccupied molecular orbital (LUMO) is computed for the uncharged state in solvent. Multiwfn software is used to obtain the HOMO and LUMO energies.

Reference

(1) Zhang, L.; Qian, Y.; Feng, R.; Ding, Y.; Zu, X.; Zhang, C.; Guo, X.; Wang, W.; Yu, G. Reversible Redox Chemistry in Azobenzene-Based Organic Molecules for High-Capacity and Long-Life Nonaqueous Redox Flow Batteries. *Nat Commun* **2020**, *11* (1), 1–11. https://doi.org/10.1038/s41467-020-17662-y.

(2) Wang, W.; Xu, W.; Cosimbescu, L.; Choi, D.; Li, L.; Yang, Z. Anthraquinone with Tailored Structure for a Nonaqueous Metal-Organic Redox Flow Batter. *Chemical Communications* **2012**, *48* (53), 6669–6671. https://doi.org/10.1039/c2cc32466k.

(3) Bartak, D. E.; Osteryoung, R. A. *The Redox Behavior of the Tetrachloro-p-Benzoquinone-Tetrachlorohydroquinone System in Molten Aluminum Chloride-Sodium Chloride Solvents*; 1976; Vol. 74.

(4) Su, C.; Yang, F.; Ji, L.; Xu, L.; Zhang, C. Polytriphenylamine Derivative with High Free Radical Density as the Novel Organic Cathode for Lithium Ion Batteries. *J Mater Chem A Mater* **2014**, *2* (47), 20083–20088. https://doi.org/10.1039/c4ta03413a.

(5) Wylie, L.; Blesch, T.; Freeman, R.; Hatakeyama-Sato, K.; Oyaizu, K.; Yoshizawa-Fujita, M.; Izgorodina, E. I. Reversible Reduction of the TEMPO Radical: One Step Closer to an All-Organic Redox Flow Battery. *ACS Sustain Chem Eng* **2020**, *8* (49), 17988–17996. https://doi.org/10.1021/acssuschemeng.0c05687.

(6) Weber, A. Z.; Mench, M. M.; Meyers, J. P.; Ross, P. N.; Gostick, J. T.; Liu, Q. Redox Flow Batteries: A Review. *J Appl Electrochem* **2011**, *41* (10), 1137–1164. https://doi.org/10.1007/s10800-011-0348-2.

(7) Kwon, G.; Lee, K.; Yoo, J.; Lee, S.; Kim, J.; Kim, Y.; Kwon, J. E.; Park, S. Y.; Kang, K. Highly Persistent Triphenylamine-Based Catholyte for Durable Organic Redox Flow Batteries. *Energy Storage Mater* **2021**, *42* (July), 185–192. https://doi.org/10.1016/j.ensm.2021.07.006.

(8) Yu, X.; Manthiram, A. Nonaqueous Hybrid Redox Flow Energy Storage with a Sodium-TEMPO Chemistry and a Single-Ion Solid Electrolyte Separator. *Energy Advances* **2022**, No. 1, 21–27. https://doi.org/10.1039/d1ya00010a.

(9) Kwon, G.; Lee, S.; Hwang, J.; Shim, H. S.; Lee, B.; Lee, M. H.; Ko, Y.; Jung, S. K.; Ku, K.; Hong, J.; Kang, K. Multi-Redox Molecule for High-Energy Redox Flow Batteries. *Joule* **2018**, *2* (9), 1771–1782. https://doi.org/10.1016/j.joule.2018.05.014.

(10) Herr, T.; Noack, J.; Fischer, P.; Tübke, J. 1,3-Dioxolane, Tetrahydrofuran, Acetylacetone and Dimethyl Sulfoxide as Solvents for Non-Aqueous Vanadium Acetylacetonate Redox-Flow-Batteries. *Electrochim Acta* **2013**, *113*, 127–133. https://doi.org/10.1016/j.electacta.2013.09.055.

(11) Korshunov, A.; Milner, M. J.; Grünebaum, M.; Grünebaum, G.; Studer, A.; Winter, M.; Cekic-Laskovic, I. An Oxo-Verdazyl Radical for a Symmetrical Non-Aqueous Redox Flow Battery †. **2020**. https://doi.org/10.1039/d0ta07891c.

(12) Hendriks, K. H.; Robinson, S. G.; Braten, M. N.; Sevov, C. S.; Helms, B. A.; Sigman, M. S.; Minteer, S. D.; Sanford, M. S. High-Performance Oligomeric Catholytes for Effective Macromolecular Separation in Nonaqueous Redox Flow Batteries. **2018**. https://doi.org/10.1021/acscentsci.7b00544.

(13) Zhang, L.; Qian, Y.; Feng, R.; Ding, Y.; Zu, X.; Zhang, C.; Guo, X.; Wang, W.; Yu, G. Reversible Redox Chemistry in Azobenzene-Based Organic Molecules for High-Capacity and Long-Life Nonaqueous Redox Flow Batteries. *Nat Commun* **2020**, *11* (1), 1–11. https://doi.org/10.1038/s41467-020-17662-y.

(14) Wang, X.; Chai, J.; Devi, N.; Lashgari, A.; Chaturvedi, A. Two-Electron-Active Tetracyanoethylene for Nonaqueous Redox Flow Batteries. **2021**. https://doi.org/10.1039/d1ta01365c.

(15) Li, Z.; Li, S.; Liu, S.; Huang, K.; Fang, D.; Wang, F.; Peng, S. Electrochemical Properties of an All-Organic Redox Flow Battery Using 2,2,6,6-Tetramethyl-1-Piperidinyloxy and N-Methylphthalimide. *Electrochemical and Solid-State Letters* **2011**, *14* (12), 5–8. https://doi.org/10.1149/2.012112esl.

(16) Gong, K.; Fang, Q.; Gu, S.; Li, S. F. Y.; Yan, Y. Nonaqueous Redox-Flow Batteries: Organic Solvents, Supporting Electrolytes, and Redox Pairs. *Energy Environ Sci* **2015**, *8* (12), 3515–3530. https://doi.org/10.1039/c5ee02341f.

(17) Yuan, J.; Zhang, C.; Zhen, Y.; Zhao, Y.; Li, Y. Enhancing the Performance of an All-Organic Non-Aqueous Redox Flow Battery. *J Power Sources* **2019**, *443* (July), 227283. https://doi.org/10.1016/j.jpowsour.2019.227283.

(18) Yurchenko, O.; Freytag, D.; Zur Borg, L.; Zentel, R.; Heinze, J.; Ludwigs, S. Electrochemically Induced Reversible and Irreversible Coupling of Triarylamines. *Journal of Physical Chemistry B* **2012**, *116* (1), 30–39. https://doi.org/10.1021/jp208076z.
